# Metabolic Regulation of the Senescence Program

**DOI:** 10.1093/geroni/igaa057.437

**Published:** 2020-12-16

**Authors:** Manali Potnis, Timothy Nacarelli, Eishi Noguchi, Ashley Azar, Christian Sell

**Affiliations:** 1 Drexel College of Medicine, Philadelphia, Pennsylvania, United States; 2 Drexel University, Philadelphia, Pennsylvania, United States

## Abstract

Cellular senescence is a cell fate defined by an irreversible cell-cycle arrest and a pro-inflammatory secretory profile. It is a consequence of a shift in metabolism and rearrangement of chromatin. Accumulation of senescent cells is a universal hallmark of age-related pathologies suggesting these cells contribute to age-related susceptibility to disease. Here, we examine the interplay between two metabolic inhibitors of senescence: Rapamycin treatment and Methionine restriction (metR). We report that a combination of methionine restriction and rapamycin induces a metabolic reprogramming that prevents activation of the senescence program in human fibroblasts. The treated cells continue to divide at a slow rate at a high passage and lack senescence-associated markers and inflammatory cytokines. Genome-wide chromatin accessibility analysis reflects chromatin remodeling with distinctly increased accessibility of heterochromatic regions in treated cells. Further, Transcriptome-wide analysis reveals increased expression of specific methyltransferases which alter the trimethylation of H3, one of the strongest hallmarks of open chromatin. This may represent a mechanistic link between a major hallmark of senescence and nuclear events required for senescence.

